# Characterization of the complete mitochondrial genome of Asia Corn Borer, *Ostrinia furnacalis* (Lepidoptera: Crambidae)

**DOI:** 10.1080/23802359.2020.1718025

**Published:** 2020-01-31

**Authors:** Chao Li, Lili Li, Yanping Ren, Zengbin Lu, Yingying Song, Li Liu, Suhong Lv, Yi Yu, Xingyuan Men

**Affiliations:** a Institute of Plant Protection, Shandong Academy of Agricultural Sciences, Shandong, Jinan, China;; b Maize Research Institute, Shandong Academy of Agricultural Science, Shandong, Jinan, China;; c College of Plant Protection, Nanjing Agricultural University, Nanjing, China;; d College of Plant Protection, Shandong Agricultural University, Shandong, Taian, China

**Keywords:** *Ostrinia furnacalis*, mitochondrial genome, phylogenetic analysis, Crambidae

## Abstract

In this study, the complete mitogenome sequence of *Ostrinia furnacalis* was described. The assembled mitogenome is 15,241 bp in length with an extreme bias of high AT content (80.9%) (GenBank accession no. MN747041). The mitochondrial genome contains 13 protein-coding genes (PCGs), 22 transfer RNA (tRNA) genes, 2 ribosomal RNA (12S rRNA and 16S rRNA) genes, and a control region (D-loop region). The mitochondrial gene order was identical to that observed in most lepidopteran genomes, nine PCGs were located on the H-strand, others were located on the L-strand. 12 PCGs were initiated by typical ATN codons, except for *COI* with CGA instead. 21 tRNAs had the typical cloverleaf structure, while the DHU arm of the *trnS1* gene did not form a stable stem-loop structure. The ‘ATAGT(A)’-like motif and a 19 bp poly-T stretch at the down-stream of the *rrnS* gene were observed in the A + T-rich region. The phylogenetic analysis showed that the relationship of *O. furnacalis* is very close to the three species in the subfamily Pyraustinae: *O. nubilalis*, *O. penitalis* and *Loxostege sticticalis*, and all the subfamilies of Spilomelinae, Pyraustinae, Crambinae and Nymphulinae within Crambidae formed monophyletic groups with the highest bootstrap value support.

The Asian Corn Borer (ACB), *Ostrinia furnacalis*, is one of the destructive lepidopteran pests of corn in Asian, and can cause significant loss in a corn field (Kojima et al. 2010). The moth larva destroys the fruit when it bores into the ear to feed on the silk and kernels, which is considered to be one of the aggravating factor for the epidemiology of *Fusarium* ear rot in maize (Folcher et al. 2009; Yang et al. 2014). Although partial mitochondrial genome sequences of *O. furnacalis* have been obtained (Coates et al. 2005), complete organelle genome information is still unclear. In the current study, the complete mitochondrial genome of *O. furnacalis* was reassembled and annotated, also the phylogenetic and taxonomic relationship with other species within Crambidae were discussed.

The larval of *O. furnacalis* selected for this study was collected from Jinan, Shandong Province, China (116°58.8′E, 36°58.7′N) in September 2017. Total genomic DNA was extracted using TIANamp Genomic DNA Kit (TIANGEN, Beijing, China) according to the manufacture’s instructions. The specimen (Voucher No. JNZQ012017) and isolated DNA were stored in the Institute of Plant Protection, Shandong Academy of Agricultural Sciences (Jinan, China). The complete mitochondrial genome of *O. furnacalis* was PCR amplified in overlapping fragments as described by Coates et al. (2005) and the fragments were assembled using MEGA6 (Tamura et al. 2013). The complete sequence was primarily annotated by MITOS WebServer (Bernt et al. 2013) and all the predicted tRNAs were confirmed using the tRNAscan-SE search server (Lowe and Chan 2016). Protein-coding genes (PCGs) and rRNA genes of *O. furnacalis* were annotated manually based on BLASTn results against published sequences of *Loxostege sticticalis* (GenBank: KR080490.1). The concatenated amino acid sequences of the 13 PCGs were used to reconstruct the phylogenetic relationships among the species within Crambidae using the Maximum Likelihood (ML) algorithm in MEGA6.0 software with the Jones–Taylor–Thornton (JTT) mode, considering 2000 replications with bootstrap analyses (Tamura et al. 2013).

The complete mitochondrial genome of *O. furnacalis* is 15,241 bp in length and has a base composition of A (41.7%), T (39.2%), C (11.4%), G (7.7%), demonstrating an extreme bias of high AT content (80.9%) (GenBank accession no. MN747041). The mitochondrial genome contains a typically conserved structure among moth mitogenomes, encoding 13 protein-coding genes (PCGs), 22 transfer RNA (tRNA) genes, 2 ribosomal RNA (12S rRNA and 16S rRNA) genes, and a control region (D-loop region). The mitochondrial gene order was identical to that observed in most lepidopteran genomes, nine PCGs were located on the H-strand, others were located on the L-strand. 12 PCGs were initiated by typical ATN codons (ATA for *ND2*, *COII* and *ATP8*; ATG for *ATP6*, *COIII*, *ND4*, *ND4L*, *CYTB* and *ND1*; ATT for *ND3*, *ND5* and *ND6*), except for *COI* with CGA instead. 21 tRNAs had the typical cloverleaf structure, while the DHU arm of the *trnS1* gene did not form a stable stem-loop structure. The ‘ATAGT(A)’-like motif and a 19 bp poly-T stretch at the down-stream of the *rrnS* gene were observed in the A + T-rich region (Chai et al. 2012). The phylogenetic analysis showed that the relationship of *O. furnacalis* is very close to the three species in the subfamily Pyraustinae: *O. nubilalis*, *O. penitalis* and *L. sticticalis*, and all the subfamilies of Spilomelinae, Pyraustinae, Crambinae and Nymphulinae within Crambidae formed monophyletic groups with the highest bootstrap value support (Figure 1).

**Figure 1.**
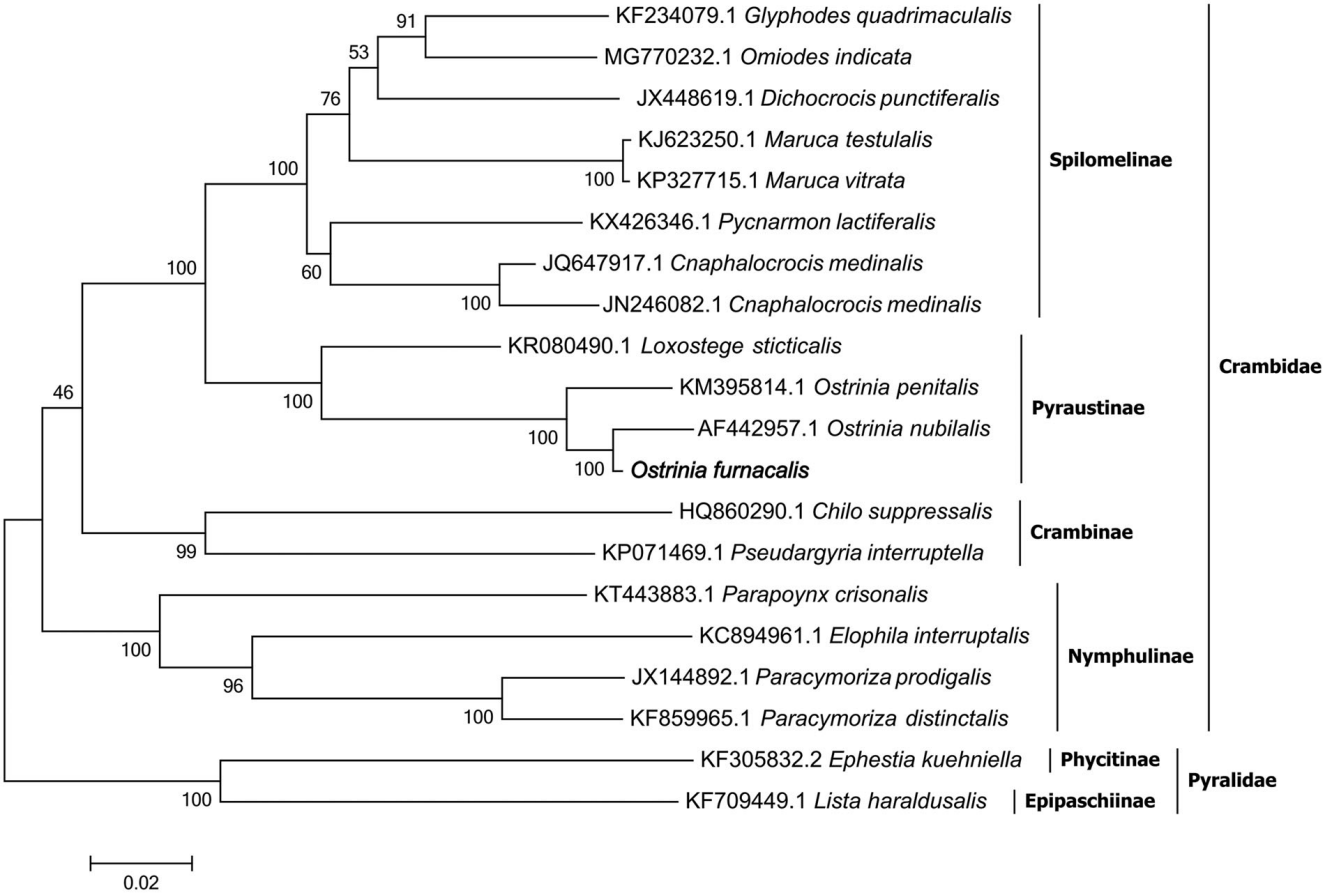
Phylogenetic tree constructed for Crambidae in Lepidoptera, including *O.furnacalis*, using the concatenated amino acid sequences of 13 PCGs. GenBank accession numbers of each species were listed in the tree. *Lista haraldusalis* and *Ephestia kuehniella* from the family Pyralidae were used as the outgroups. The tree was constructed based on a complete protein sequence alignment by the ML method with bootstrapping analysis (2000 replicates).
